# Physiotherapy interventions encouraging frequent changes of the body position and physical activity for infants hospitalised with bronchiolitis: an internal feasibility study of a randomised control trial

**DOI:** 10.1186/s40814-022-01030-2

**Published:** 2022-03-30

**Authors:** Sonja Andersson-Marforio, Annika Lundkvist Josenby, Christine Hansen, Eva Ekvall Hansson

**Affiliations:** 1 Department of Health Sciences, Lund University, Margaretavägen 1B, S-22240 Lund, Sweden; 2 Children’s Hospital, Skåne University Hospital, S-22185 Lund, Sweden

**Keywords:** Feasibility studies, Physical therapy modalities, Bronchiolitis, Randomised controlled trials

## Abstract

**Background:**

The effect of a treatment that includes frequent changes of the body position for infants with bronchiolitis has not been evaluated, although it is often used in Swedish hospitals. Because of this, a randomised control trial (RCT) has begun with the aim to evaluate this treatment, comparing the effect of an individualised physiotherapy intervention, a non-individualised intervention, and standard care in a control group. The objective of this internal pilot study was to address uncertainties concerning the ongoing RCT and to determine whether the trial is feasible or not, possibly with adjustments to the protocol.

**Methods:**

Descriptive analyses of the recruitment, retention, data supply for the primary end point, and the usability of the primary outcome measure in the full RCT were performed. A safety analysis was conducted by an independent analysis group.

**Results:**

Ninety-one infants were included, 33 (36.3%), 28 (30.8%), and 30 (33.0%) in the respective allocation groups. Fifty-nine (64.8%) were boys. The median age was 2.5 (min–max 0.2–23.7) months. They remained in the study for a median of 46 hours (min–max 2–159). The recruitment rate was 19%. The data supply for the primary end point and for the primary outcome measure was lower than anticipated in the original sample size calculation. Difficulties concerning utilising the primary outcome measure were identified. The safety analysis detected no risks of harm related to participation in the study.

**Conclusions:**

It is feasible to continue the RCT with modifications of the analysis plan. Participation in the study was not associated with any safety risks.

**Trial registration:**

ClinicalTrials.gov NCT03575091. Registered 2 July 2018. Retrospectively registered.

**Supplementary Information:**

The online version contains supplementary material available at 10.1186/s40814-022-01030-2.

## Key messages regarding feasibility

- The main uncertainties of the full RCT concerned recruitment, retention, data collection, the primary outcome measure, and the safety for participants.
- The recruitment and the retention rates were low, which affected the data supply at the chosen time of analysis. The primary outcome measure was not feasible for use in the full RCT and will be changed. No harmful outcome was detected.
- It is feasible to continue the RCT with some adjustments to the analysis protocol. It is safe to participate in the study.

## Background

Lower respiratory tract infections such as bronchiolitis or pneumonia is the most common reason for infants around the world to become hospitalised [1]. The reason for hospitalisation is often respiratory distress following increased mucus production and oedema in the smaller airways, and subsequent feeding difficulties [2, 3]. The treatment in hospitals is mostly supportive, and most patients are treated with supplemented oxygen and fluid [4], and the use of high flow nasal cannula (HFNC) is extensive [5]. Some infants may need treatment at an intensive care unit (ICU) [6]. To support evacuation of bronchial secretion, reduce respiratory distress and increase oxygenation, physiotherapy (PT) treatment is sometimes used [7–10].

The evidence about the effect of PT treatment for infants hospitalised with acute bronchiolitis or pneumonia is unclear [11]. Furthermore, PT treatment defined as vibration, percussion, postural drainage, slow passive expiratory techniques, and forced passive expiratory techniques is not generally recommended [12]. Given the great variety in the described PT treatment methods and their evaluation methods, guidelines about ‘chest physiotherapy’ in general are not applicable for all PT treatment methods. Our research group conducted a survey study to understand and describe current Swedish practices [13], which comprise different methods, but usually with a focus on frequent changes of the body position and stimulation of physical activity. To our knowledge, this treatment for the patient group has not previously been described or evaluated.

To evaluate this treatment, only recently described for infants with acute respiratory infections in hospitals [13], the research group started a randomised control trial (RCT). The main purpose of the RCT is to evaluate the effect of this PT treatment in two intervention groups compared to a control group receiving standard care. The details of the RCT are described in a study protocol [14]. There were, however, uncertainties about the feasibility of completing the RCT which we decided to evaluate in this study, as is also recommended in the Medical Research Council’s (MRC) guidelines on complex interventions [15] and that are further explained by Craig et al. [16] and Richards and Hallberg [17]. They stress that it is important to acknowledge and to structure the research process in different steps in order to produce valid results. In the feasibility and piloting step, according to the MRC’s guidelines and others [15, 18], the researchers test the procedures, the outcome measure, estimate recruitment and retention, and examine the eligibility criteria. Hence, in order to produce high quality research, and in the light of a well-designed study being less likely to produce research waste [19], testing the feasibility of the full RCT was warranted. The aim of this study was to address uncertainties concerning the ongoing RCT and to determine whether the trial is feasible or not, or what adjustments to the protocol are needed.

## Methods

We aimed at making a critical analysis of the feasibility of the protocol concerning recruitment, retention, the chosen point for the primary analysis, and the primary outcome measure, as well as perform a safety analysis, following the intention of the study protocol [14].

### Trial design

This is an internal pilot study [20] conducted in an ongoing clinical two-centre individually randomised controlled trial with three parallel groups. We designed the study with inspiration from literature on the subject [15, 18, 21, 22]. The results are reported using the CONSORT extended guideline for pilot and feasibility trials [23], as recommended by Thabane et al. [24].

### Inclusion of participants

The participants were identified and recruited by the staff in the paediatric wards of two hospitals in the south of Sweden. Inclusion criteria were as follows: age 0–24 months, hospitalised on the basis of acute airway infection, born in gestational week 35 or later. Patients had to be included within 24 hours (h) of hospital admission. At least one of the parents/guardians had to understand written Swedish, English, Arabic, or Persian. Exclusion criteria: previous respiratory or cardiac diagnoses. The participants were included between November 2017 and March 2020. The seasonal inclusion period was typically between November and April. In order to admit their child into the study, the parent/s signed a written consent form. The participation in the study ended when the infants were either discharged to home, referred to an ICU or when the parents decided to withdraw the participation of their child.

### Interventions

Details about the interventions are provided in the study protocol [14]. The participants were randomised to an individualised physiotherapy intervention, a non-individualised intervention, or a control group. A statistician independent of the research group performed the randomisation, stratified by the two sites, and prepared opaque paper envelopes. The staff in care of the recruited participant at the ward opened the top envelope in the study binder to reveal the allocation group.

All three groups received the standard care at the ward, and the two intervention groups received additional treatment, including different movements of the body. The standard care comprised information to the parents about the importance of fluid intake for their infant, oxygen supplementation, nose drops and suctioning, high flow nasal cannula (HFNC), inhalations, fluid supplementation, and analgesics, according to need. The individualised intervention was performed by a physiotherapist at least once daily. The PT was sitting on a large ball, firmly supporting the infant in different body positions, while bouncing, in order to affect the respiratory pattern of the infant: increase the expiratory air flow and stimulate deep inspirations. The PT also stimulated active movements according to the infant’s ability and could choose additional treatments. The non-individualised intervention was performed by the nursing staff at least once shortly after inclusion and comprised changes of the body position mainly out the bed, but not using the ball, and slightly less variation of activities. After the first 20-min intervention, the parents in both intervention groups were instructed to continue the movements regularly throughout the day.

### Outcomes

Assessments were made at baseline, after 20 minutes (directly following the first intervention), and every subsequent third hour. The primary outcome measure in the RCT is a composite index that the research group constructed with scores from 0 (worst condition) to 11, based on factors that determine if an infant needs hospitalisation [25]. The composite index comprises levels of oxygen saturation, supplemented oxygen concentration, high nasal flow treatment, and oral fluid intake (as opposed to tube feeding).

The secondary outcome measures include the Wang score [26], standard vital signs such as heart rate, the parents’ observations (on general condition and food intake), time spent at the hospital ward, and referrals to an intensive care unit.

According to the analysis plan for the full RCT, baseline assessments will be compared with the assessments after 24 h. We have also planned to examine any immediate effect of the first intervention, after 20 minutes.

*The recruitment* was analysed in four different ways: the proportion of included participants, the estimated time to reach target sample size, time to inclusion, and possible improvement before inclusion.

The proportion of included participants was assessed by comparing the number of participants included in the study with the number of patients admitted to the wards during the three winter seasons 2017–2018, 2018–2019, and 2019-2020 recorded with the International Statistical Classification of Diseases and Related Health Problems - Tenth Revision (ICD-10) diagnoses of respiratory infections J10.0, J11.0, J12.0, J13.0, J14.0, J15.0, J18.0, and J21.0 with subgroups*.* We additionally assessed what proportion of the infants admitted to one of the hospital wards with the diagnoses above met the inclusion criteria, by reading the medical records of admitted patients at four weeks during the peaks of the Respiratory Syncytial Virus (RSV) infection in Sweden according to national reports [27]. The selected weeks were February 26 to March 4 and March 19 to 25 in 2018 and February 11 to 24 in 2019. We further estimated how long it would take to include 162 patients (primary sample size calculation) in the study, based on how many had been included during these three winter seasons.

Time to inclusion was calculated by comparing the time for admission to the ward (recorded in the local patient administrative system) to the time for the baseline assessment. This item was reported in hours.

A possible improvement before inclusion was analysed to examine the inclusion criteria of admitted delay for 24 h after admittance to the ward. The possibility to detect any changes from baseline and between groups may decline with increasing time if the participants might already have recovered substantially before inclusion. This was analysed as follows: the infants included after the median time to inclusion were further analysed. Those who received oxygen supplementation or HFNC at the first assessment were excluded as they were considered still severely affected, and the delay was thus judged as acceptable*.* For the infants who did not have oxygen supplementation or HFNC at inclusion, we compared levels of oxygen saturation, respiratory rate, and heart rate at admittance to the ward with values at inclusion. If the values had changed from one level to another in the Rapid Emergency Triage and Treatment System–pediatric (RETTS-p) [28–31], which is commonly used in Swedish hospitals, the change was considered as clinically significant and more problematic for the study. Additionally, and in the same way, we analysed change in RETTS-p level among the infants who were included by mistake after 24 h at the ward, and who thus did not fulfill the inclusion criteria.

*Retention* was primarily analysed by calculating how many hours the participants remained in the study. We further analysed the proportion of participants still in the study with recorded data about saturation or heart rate at hour 24 (primary end point), hour 36, hour 48, and longer. We analysed data from these occasions to examine participant retention at the chosen point for analysis, and the feasibility of delaying the primary end point. Our theory was that the possibility to detect changes from baseline and analyse any differences between the groups would improve the closer to discharge the analysis was made. Based on the previous sample size calculation and the planned analysis for the full RCT, a retention rate of 85% or more was considered acceptable.

### Primary outcome measure

The proportion of complete reported data in the composite index at baseline and at hour 24 was analysed using frequency measures. Each of the four domains in the index was analyzed separately, i.e. level of oxygen saturation, concentration of supplemented oxygen, level of supplied high nasal air flow, and oral fluid intake as a proportion of calculated daily need. A level of 85% was determined as sufficient, following the previous sample size calculation. We additionally critically reviewed the quality of the collected data in the composite index in order to determine usability.

*The safety analysis* was performed by an independent group consisting of a statistician and a paediatrician. Following the instructions from the research group, they thoroughly reviewed the data, searching for values that in a clinical setting would necessitate extra treatment or dramatically change the treatment for an infant. The values included in the safety analysis were oxygen saturation, respiratory rate, oxygen supplementation, high nasal air flow, oral fluid intake, length of hospital stay, referrals to ICU, and deaths. They analysed values from the total study population. If values indicating any safety risks had been discovered, they would have continued by analysing the data divided into the separate intervention groups. If they had identified any safety risks, the research group would, after clinical reasoning, either have changed the protocol or terminated the study.

### Sample size and statistical methods

The sample size calculation for the full RCT showed that 162 participants need to be included, 54 in each group. Safety and interim analyses were planned to be performed after inclusion of 50% of the required participants, and this feasibility study was performed at this stage accordingly [14]. The safety analysis group was blinded regarding allocation group for the participants. Blinding of parents to participants, care providers, or assessors was not possible due to the nature of the intervention. To minimise possible bias, one person performed the intervention, and another person made the assessment.

In this feasibility study we used the IBM SPSS Statistics 27 Windows (IBM Corporation, Armonk, NY, USA) for analyses of the data. The analyses were descriptive, using median with min–max (IQR), and numbers with percentages when appropriate.

## Results

**Fig. 1:**
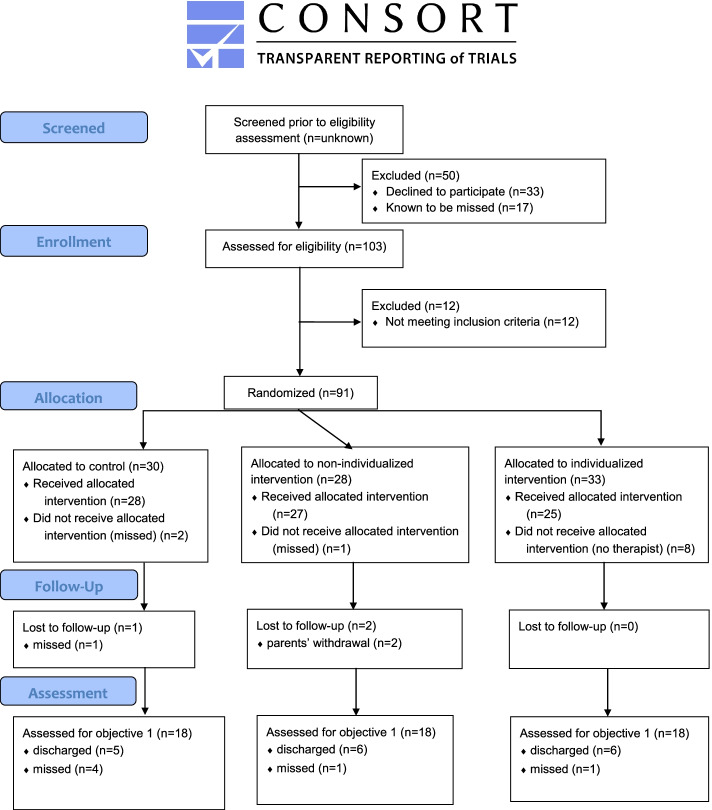
Flow chart of the participant flow

For a description of the participant flow, see Fig. 1. Of the 91 included participants, 11 did not start any intervention or assessment for different reasons and were thus drop-outs after randomisation.

Table 1 displays participants’ baseline characteristics

**Table 1:** Baseline characteristics of the included participants, *n* = 91

	Control group ***n*** = 30	Non-individualised intervention ***n*** = 28	Individualised intervention ***n*** = 33	Total sample ***n*** = 91
**Gender** *n* (%) Male/female	21 (67.7)/9 (30.0)	15 (54.6)/13 (46.4)	23 (69.7)/10 (30.3)	59 (64.8)/32 (35.2)
**Age** median (min–max) Months	3.3 (0.2–22.4)	2.9 (0.5–22.2)	2.1 (0.3–23.7)	2.5 (0.2-23.7) IQR^a^ 1.2, 6.9
**Infectious agent** *n* (%)^b^				
RSV^c^	18 (60.0)	19 (67.9)	22 (66.7)	59 (64.8)
Influenza	2 (6.7)	3 (10.7)	4 (12.1)	9 (9.9)
Other	1 (3.3)	3 (10.7)	3 (9.1)	7 (7.7)
Negative	5 (16.6)	1 (3.6)	1 (3.0)	7 (7.7)
Missing	5 (16.6)	2 (7.1)	4 (12.1)	11 (12.1)
**Heredity atopic disease** *n* (%)				
Asthma	10 (33.3)	10 (35.7)	12 (36.4)	32 (35.2)
Other (allergies, eczema etc.)	7 (23.3)	5 (17.9)	11 (33.3)	23 (25.3)
None	10 (33.3)	10 (35.7)	3 (9.1)	23 (25.3)
Missing	3 (10.0)	3 (10.7)	7 (21.2)	13 (14.3)
**Passive smoking** exposure *n* (%)				
No	22 (73.3)	21 (75.0)	17 (51.6)	60 (65.9)
Yes	5 (16.6)	3 (10.7)	8 (24.2)	16 (17.6)
Missing	3 (10.0)	4 (14.3)	8 (24.2)	15 (16.5)

^a^*IQR* Interquartile range

^b^Two individuals had positive tests for both RVS and influenza

^c^RSV Respiratory syncytial virus

Outcomes of the feasibility study are reported below.

### Recruitment

During the stated 4 weeks in 2018 and 2019, 46 infants were hospitalised with the selected diagnoses of respiratory infections. Twenty-nine infants (63%) met the inclusion criteria and 17 (37%) did not, due either to age (*n* = 12), heart disease (*n* = 2), asthma (*n* = 2), or premature birth (*n* = 1). During the entire study period, 762 infants were admitted to the two wards with the selected diagnoses of respiratory infections. By using the results from the in-depth examination above on the whole sample (63% estimated to meet the inclusion criteria), 480 patients remained eligible. During the study period, 91 infants were included, which constitutes an inclusion rate of 19%. Parents of 33 infants (7%) were recorded to have declined participation. The number of parents not asked to participate is not known in detail, but the number of those recorded to have been missed is displayed in the flow chart in Fig. 1.

In the winter season 2017–2018, 33 participants were included, in the season 2018–2019, 27 participants, and in the season 2019–2020, 31 participants. If the inclusion rate follows the same pace, an additional 2.5 winter seasons will be needed to reach the original calculated sample size.

For the 80 infants who started assessments and interventions, participation began at median 13 hours (min–max 0–24 h, IQR 6, 18) after admission to the hospital wards.

Twenty-three infants without supplemented oxygen or HFNC were included after 13 h and were analysed regarding vital signs. Six of them had improved regarding RETTS-p before inclusion in the study, and one had deteriorated. Individual data are available in Additional file 1. We additionally analysed 11 individuals who were excluded from the first analysis as they did not meet the inclusion criteria of being included within 24 h. They were included after a median of 31 hours (min–max 25–36 h). We omitted four infants who received oxygen supplementation or HFNC at inclusion, and seven remained. Six of these had improved and one had remained on the same RETTS-p level. Individual data are available in Additional file 2. Six of 91 infants in the study and six of 11 who were included after 24 hours were shown to improve significantly before inclusion.

### Retention and time for analysis

The participants remained in the study for a median of 46 hours (min–max 2–159 h, IQR 22, 71), calculated on the 80 participants who started their intervention/control.

The proportions of all included participants (*n* = 91) still in the study with data on saturation or heart rate at 24, 36, and 48 h are displayed in Table 2. For 33 infants (36.3%) some incomplete data was also reported after 48 h.

**Table 2:** Participant retention and data supply at different times for follow-up, *n* = 91

	24 h ***n*** (%)	36 h ***n*** (%)	48 h ***n*** (%)
**Saturation**			
Valid	57 (62.6)	42 (46.2)	35 (38.5)
Discharged	16 (17.6)	25 (27.5)	32 (35.2)
Drop-outs^a^	13 (14.3)	13 (14.3)	13 (14.3)
Missed^b^	5 (5.5)	11 (12.1)	11 (12.1)
Still in the study^c^	62 (68.1)	52 (57.1)	46 (50.5)
**Heart rate**			
Valid	57 (62.6)	43 (47.3)	34 (37.4)
Discharged	16 (17.6)	25 (27.5)	32 (35.2)
Drop-outs^a^	13 (14.3)	13 (14.3)	13 (14.3)
Missed^b^	5 (5.5)	10 (11.0)	12 (13.2)
Still in the study^c^	62 (68.1)	52 (57.1)	46 (50.5)

^a^Drop-outs indicates infants who either did not start the interventions at all (*n* = 11), or infants for whom the parents withdrew their participation after some time (*n* = 2)

^b^‘Missed’ indicates that the score was not filled out at this specific time, but the patient was still at the ward and there are values possible to impute from the assessments before and/or after this

^c^‘Still in the study’ comprises the combined values for ‘valid’ and ‘missed’, i.e. recorded data or values possible to impute

At 24 hours, there was less data recorded than needed for the primary analysis based on earlier sample size calculation, and data continued to be lost over increasing time.

### Primary outcome measure

The proportions of complete reported data in the composite index for the participants who started interventions/controls (*n* = 80) are displayed in Table 3.

**Table 3:** Proportion of complete reported data and missing data in the composite index (primary outcome measure), *n* = 80

	Complete ***n*** (%)	Missing ***n*** (%)
**Level of oxygen saturation**		
At baseline	80 (100.0)	0 (0.0)
At 24 h	57 (71.3)	23 (28.7)^a^
**Concentration of supplemented oxygen**		
At baseline	61 (76.3)	19 (23.8)^b^
At 24 h	43 (53.8)	37 (46.3)^c^
**Level of supplied high nasal flow treatment**		
At baseline	80 (100.0)	0 (0.0)
At 24 h	54 (67.5)	26 (32.5)
**Oral fluid intake**		
The first 24 h	25 (31.3)	55 (68.8)^d^

^a^Due to discharged, missed registration, or drop-outs

^b^Due to receiving low flow O_2_ or missed registration

^c^Due to low flow O_2_ supplementation, discharged, missed registration, or drop-outs

^d^Due to incomplete observations, missed registration, or discharged

At the wards, the oxygen saturation was recorded either with or without oxygen supplementation, which makes it difficult to estimate the severity of illness using the scoring, and when removing participants without supplemented oxygen there was a low level of complete recorded data on this variable (38.8%) on the two occasions. At baseline, 32 individuals (40%) received oxygen supplementation, and at 24 hours 24 (30%) did.

The concentration of oxygen supplementation in the composite index is based on high flow oxygen supplementation and does not include participants with low flow oxygen supplementation. Of the 80 participants who started interventions or controls, 18 (22.5%) received low flow oxygen supplementation at the first assessment. At 24 hours, 12 of the 80 (15.0%) received low flow oxygen supplementation*.*

The proportion of complete recorded data for the primary outcome is lower than anticipated when the sample size for the RCT was calculated. The registration of oxygen saturation for participants both with and without supplemented oxygen, as well as the use of high and low flow oxygen supplementation at the wards, further complicates the use of the composite index.

### Adjustments for the full RCT

Based on the analyses and data from this study, for which see also Additional file 3, we have decided to change the primary outcome measure to ‘time to improvement’, which is defined as the time before one of the following events occurs: reduced total Wang respiratory score, ceased use of supplemented oxygen, ceased use of supplemented HFNC, ceased use of gastric tube for feeding, or discharge to the home. We have also decided to change the statistical analysis method to a survival analysis (time-to-event). A new sample size calculation was performed based on the minimal clinically important difference to be 3 hours. The power probability was determined to 80%, with a significance level of 0.025. For three groups of equal size, 40 participants in each group were needed for the two intervention groups to be compared to the control group, making a total of 120 participants.

### The safety analysis

The safety analysis group reported no adverse events or values indicating any safety risks associated with participation in the study when analysing the entire study population. Thus, they did not continue to analyse the data divided into the respective intervention groups.

## Discussion

This study was conducted with the aim of assessing the feasibility of on ongoing RCT that will evaluate the common PT praxis in Sweden involving frequent changes of the body position for infants hospitalised with bronchiolitis or other lower respiratory tract infections. Our results show that the data supply for the primary end point and for the primary outcome measure was lower than anticipated in the original sample size calculation. Difficulties concerning utilising the primary outcome measure were identified. The safety analysis detected no risks of harm related to participation in the study. We agree with Chalmers et al. [32] that for scientific and ethical reasons it is important that clinical research is well designed, as it is performed using public funds and involves many people, in this case infants with respiratory infections, their parents, and busy nursing staff, and this study has contributed to increase the feasibility of an ongoing trial.

### Objectives

Our main concerns were whether the recruitment, retention, primary outcome measure, and follow-up time for analysis were feasible for the RCT, and we also wanted to assess possible safety risks.

The outcome of an intervention in an acute hospital setting is not always easy to establish, as is also discussed elsewhere [33, 34]. The primary outcome measure in the RCT was chosen because of its clinical implication and objectivity. We were not certain, however, about the usability of the composite index and whether it was possible to collect the data successfully. Further, we did not know how long the participants would stay hospitalised. In order to capture as much improvement data as possible, we wanted to make the primary analysis as close to the infants’ discharge as possible, so looked for the most appropriate time for analysis. In order not to miss any major clinical improvement before inclusion, we also wanted to analyse the admitted time delay before inclusion.

### Recruitment

We did not find previous data on anticipated recruitment rate suitable for this study, and our assessment of the outcome is based on clinical reasoning. We expected many infants to be pre-terms or to have comorbidities, and thus not fulfill the inclusion criteria. The data in this study partly support this theory, but the recruitment rate is even lower than we expected, based on clinical reasoning about the high prevalence of infants with bronchiolitis in hospitals. Some parents obviously rejected the offer to participate in the study, and the reasons for that remain to be studied. However, the most likely contributory factor to the low recruitment rate was that many parents were not asked to participate. Many infants with respiratory diagnoses are hospitalised during the night, and we have received informal information from the staff that they were reluctant to ask for participation in the study at those hours so as not to disturb the families or because of working routines. During peaks of the RSV infection, the staff also expressed that they were sometimes too busy to enrol participants.

Some of the infants included in the study within the stipulated time showed a clinically significant improvement before the interventions started, and of the infants included after 24 h a larger proportion improved before inclusion. This supports our view that it would have been preferable if all participants had been included immediately after admittance to the ward, which unfortunately may prove difficult in clinical reality, as this study has shown. The results in the full RCT may be affected, though in this case to a rather limited extent.

### Retention

This study showed that the hospital stay was short, which, together with the drop-outs and missed registrations, resulted in low data supply. These findings were important for us when considering the continuation of the full RCT and may also be interesting for other researchers planning to undertake similar clinical studies. We did not fully anticipate the proportion of missed registrations, as the assessment protocol was constructed together with nursing staff to make it easy to fill out. In order to enhance recruitment and data collection it would probably be helpful to have extra staff present on the sites at all times with the responsibility to recruit participants and support data collection. The analyses in this study on participant retention and data supply do not support a primary analysis at 24 hours, and it will not be feasible to postpone the time for the primary analysis.

### Primary outcome measure

Data for the composite index in total was considerably lacking. The data on HFNC and oxygen saturation, however, was complete for all participants in the study at baseline, and ample data supply was retained at hour 24, so it was feasible to collect these items rather successfully. There was a considerable lack of data in the feeding score, which was somewhat surprising to the research group. For infants who were breast-fed, we learned that the practice at the wards was often to encourage the mothers themselves to put the infant on the scales before and after feeding and note the different weights. This is understandably difficult to undertake at all hours, not least during the night, and might have contributed to the low level of data. There may also have been a pedagogical gap towards the staff about the importance of recording this item properly, as they sometimes marked the paper protocols with an X followed by the explanatory note “breast feeding”. Because of the low data supply and the difficulties in utilising the composite index, the primary outcome measure for the full RCT will be changed.

### Safety analysis

We agree with Ioannidis et al. [35] about the importance of assessing and reporting harms in clinical trials. As no safety risks were identified in this study, we do not anticipate any harm to be connected to completing the full RCT.

### Adjustments for the full RCT

Since this study revealed issues related to feasibility that needed to be improved, some changes for the full RCT were suggested. The proposed change of primary outcome measure and analysis method has the advantage of capturing improvement through the entire hospital stay and will thus not be restricted to one pre-set time (previous 24 h), which is supported by data in this study. Moreover, the new sample size calculation, assuming less participants than originally planned, is favourable considering the low recruitment rate. Taken together, these changes will support the feasibility to complete the trial successfully.

### Limitations

The number of patients screened was unknown and a screening log would have been of benefit. It was difficult to determine progression criteria or cut-off points for the different outcomes, which is desirable for feasibility studies [19]. We have, however, tried to make informed clinical reasoning around this, to compensate for the lack of external guidance. Adding interviews with staff might have provided further information about the difficulties concerning recruitment and data recording, and possibly also pointed towards solutions for these. However, the first author (SAM) has regularly received informal information, although not scientifically structured, from meetings with the contact staff, the management and with all staff together while running the trial.

## Conclusions

It is feasible to continue the full RCT with modifications of the analysis plan. As the study concerns treatment for a large and vulnerable group of patients, it is valuable for clinical as well as ethical reasons to make use of the collected data, continue the ongoing RCT, and evaluate the effect of the PT interventions. Participation in the study was not associated with any safety risks.

## Supplementary Information

**Additional file 1. **Change in RETTS-p score before enrolment in the study for participants included after the median time to inclusion and without O_2_ supplementation or HFNC, *n*=23.

**Additional file 2. **Change in RETTS-p score before enrolment for infants included after 24 hours and without O_2_ supplementation or HFNC, *n*=7.

**Additional file 3.** Table of data supply at the different assessments in the study for n individuals.

## Data Availability

All data are archived according to the Swedish Act concerning the Ethical Review of Research Involving Humans to attain confidentiality and are available from the corresponding author on reasonable request.

## Abbreviations

RCT: Randomised control trial
RSV: Respiratory syncytial virus
HFNC: High flow nasal cannula
ICU: Intensive care unit
PT: Physiotherapy/physiotherapist
h: Hours
ICD-10: International Statistical Classification of Diseases and Related Health Problems-Tenth Revision
RETTS-p: Rapid Emergency Triage and Treatment System–pediatric
IQR: Interquartile range
