# Correction: Physiotherapy interventions encouraging frequent changes of the body position and physical activity for infants hospitalised with bronchiolitis: an internal feasibility study of a randomised control trial

**DOI:** 10.1186/s40814-022-01063-7

**Published:** 2022-05-19

**Authors:** Sonja Andersson-Marforio, Annika Lundkvist Josenby, Christine Hansen, Eva Ekvall Hansson

**Affiliations:** 1 Department of Health Sciences, Lund University, Margaretavagen 1B, S-22240 Lund, Sweden; 2 Children’s Hospital, Skane University Hospital, S-22185 Lund, Sweden

**Correction: Pilot Feasibility Stud 8, 76 (2022)**

**https://doi.org/10.1186/s40814-022-01030-2**

Following publication of the original article [1], the authors reported an error in the heading “Primary outcome measure” found on page 4. The heading should be at the same level as “*The safety analysis*” further down and “*The recruitment*” and “*Retention*” on the page before under the heading Outcomes.

The original article [1] has been updated.
