# Supplementary material for: Physiotherapy interventions encouraging frequent changes of the body position and physical activity for infants hospitalised with bronchiolitis: an internal feasibility study of a randomised control trial
Source: Pilot Feasibility Stud. 2022 Mar 30;8:76. doi: 10.1186/s40814-022-01030-2 (PMC8966163; doi:10.1186/s40814-022-01030-2)
Supplement: Supplementary file 1 — Additional file 1. Change in RETTS-p score before enrolment in the study for participants included after the median time to inclusion and without O2 supplementation or HFNC, n=23. [file 40814_2022_1030_MOESM1_ESM.docx]

Additional file 1. Change in RETTS-p score before enrolment in the study for participants included after the median time to inclusion and without O_2_ supplementation or HFNC, n=23

| **Sex** | **Age, months** | **Change RETTS-p*** | **Time before enrolment, hours** |
| --- | --- | --- | --- |
| boy | 1.4 | **-** | 20.75 |
| boy | 22.1 | HR orange → yellow, RR orange → yellow | 21.08 |
| girl | 1.2 | - | 15.00 |
| boy | 9.6 | HR yellow → green | 17.38 |
| girl | 13.5 | Sat yellow → green, HR red → yellow, RR missing | 17.25 |
| boy | 17.6 | HR orange → yellow, sat orange → yellow, RR red → green | 15.00 |
| girl | 7.3 | - | 24.17 |
| girl | 1.7 | - | 15.50 |
| boy | 1.4 | - | 20.17 |
| boy | 1.3 | - | 19.42 |
| boy | 1.3 | - | 14.20 |
| girl | 2.9 | - | 23.67 |
| girl | 2.5 | - | 19.18 |
| boy | 16.1 | HR yellow → green | 20.50 |
| boy | 3.1 | - | 14.50 |
| boy | 15.7 | Sat orange → green | 19.08 |
| girl | 10.1 | - | 13.75 |
| boy | 5.1 | - | 20.17 |
| boy | 12.3 | Sat green → orange, RR green → yellow | 15.17 |
| girl | 0.8 | - | 13.25 |
| girl | 2.8 | - | 16.50 |
| boy | 0.9 | - | 14.07 |
| boy | 2.0 | - | 13.17 |

*Colours indicating priority levels of RETTS-p for examination by a physician in an emergency department after basic evaluation and treatment: green=“can wait a maximum of 4 hours”, yellow=“can wait a maximum of 2 hours”, orange=“potentially life threatening, examination within 20 minutes, red=“life threatening, urgent physician examination”
HR=heart rate, RR=respiratory rate, Sat=oxygen saturation
