# Supplementary material for: Physiotherapy interventions encouraging frequent changes of the body position and physical activity for infants hospitalised with bronchiolitis: an internal feasibility study of a randomised control trial
Source: Pilot Feasibility Stud. 2022 Mar 30;8:76. doi: 10.1186/s40814-022-01030-2 (PMC8966163; doi:10.1186/s40814-022-01030-2)
Supplement: Supplementary file 2 — Additional file 2. Change in RETTS-p score before enrolment for infants included after 24 hours and without O2 supplementation or HFNC, n=7. [file 40814_2022_1030_MOESM2_ESM.docx]

Additional file 2. Change in RETTS-p score before enrolment for infants included after 24 hours and without O_2_ supplementation or HFNC, n=7

| **Sex** | **Age,  months** | **Change RETTS-p*** | **Time before inclusion, hours** |
| --- | --- | --- | --- |
| boy | 19.54 | Sat orange → green | 26.00 |
| boy | 0.39 | - | 33.00 |
| girl | 1.78 | HR orange → yellow | 30.78 |
| girl | 18.42 | RR yellow → green, HR yellow → green | 25.25 |
| girl | 16.84 | HR yellow → green | 25.58 |
| girl | 7.63 | RR yellow → green | 25.75 |
| girl | 7.53 | Sat orange → green | 34.00 |

*Colours indicating priority levels of RETTS-p for examination by a physician in an emergency department after basic evaluation and treatment: green=“can wait a maximum of 4 hours” 2=yellow “can wait a maximum of 2 hours”, orange=“potentially life threatening, examination within 20 minutes, red=“life threatening, urgent physician examination”
Sat=oxygen saturation, HR=heart rate, RR=respiratory rate
