# Supplementary material for: Physiotherapy interventions encouraging frequent changes of the body position and physical activity for infants hospitalised with bronchiolitis: an internal feasibility study of a randomised control trial
Source: Pilot Feasibility Stud. 2022 Mar 30;8:76. doi: 10.1186/s40814-022-01030-2 (PMC8966163; doi:10.1186/s40814-022-01030-2)
Supplement: Supplementary file 3 — Additional file 3. Table of data supply at the different assessments in the study for n individuals. [file 40814_2022_1030_MOESM3_ESM.docx]

Additional file 3. Table of data supply at the different assessments in the study for n individuals.

| Assessment at hour, h | Wang  total score |  | Oxygen suppl |  | HFNC |  | Hospital stay |  |
| --- | --- | --- | --- | --- | --- | --- | --- | --- |
|  | valid n | missing* n | valid  n | missing n | valid n | missing n | valid  n | missing n |
| baseline, 0 h | 76 | 15 | 81 | 10 | 82 | 9 | 82 | 9 |
| 20 minutes | 70 | 21 | 76 | 15 | 77 | 14 | 80 | 11 |
| 3 h | 66 | 25 | 77 | 14 | 77 | 14 | 80 | 11 |
| 6 h | 55 | 36 | 67 | 24 | 67 | 24 | 80 | 11 |
| 9 h | 51 | 40 | 62 | 29 | 62 | 29 | 80 | 11 |
| 12 h | 47 | 44 | 58 | 33 | 57 | 34 | 79 | 12 |
| 15 h | 47 | 44 | 57 | 34 | 56 | 35 | 78 | 13 |
| 18 h | 52 | 39 | 61 | 30 | 61 | 30 | 78 | 13 |
| 21 h | 47 | 44 | 55 | 36 | 54 | 37 | 78 | 13 |
| 24 h | 50 | 41 | 91 | 0 | 84 | 7 | 78 | 13 |
| 27 h | 44 | 47 | 49 | 42 | 45 | 46 | 78 | 13 |
| 30 h | 42 | 49 | 48 | 43 | 47 | 44 | 78 | 13 |
| 33 h | 34 | 57 | 42 | 49 | 40 | 51 | 78 | 13 |
| 36 h | 31 | 60 | 43 | 48 | 42 | 49 | 78 | 13 |
| 39 h | 36 | 55 | 44 | 47 | 44 | 47 | 78 | 13 |
| 42 h | 38 | 53 | 40 | 51 | 40 | 51 | 78 | 13 |
| 45 h | 32 | 59 | 36 | 55 | 35 | 56 | 78 | 13 |
| 48 h | 33 | 58 | 36 | 55 | 36 | 55 | 78 | 13 |

*missing=drop-outs (at baseline 9, after 20 minutes additionally 2, later additionally 2=13 in total), discharged, missed registration

Additional file 3. Table of data supply whether receiving tube feeding or not, n individuals

| Time, hours | Valid n | Missing* n |
| --- | --- | --- |
| First 24 | 79 | 12 |
| 25-48 | 67 | 24 |
| 49-72 | 45 | 46 |
| 73-96 | 24 | 67 |
| 97-120 | 16 | 75 |

*missing = drop-outs (11 the first 24 h, later 2 additional=13), discharged, missed registration
